# A systematic review and meta-regression of the knowledge, practices, and training of restaurant and food service personnel toward food allergies and Celiac disease

**DOI:** 10.1371/journal.pone.0203496

**Published:** 2018-09-04

**Authors:** Ian Young, Abhinand Thaivalappil

**Affiliations:** 1 School of Occupational and Public Health, Ryerson University, Toronto, Ontario, Canada; 2 Department of Population Medicine, University of Guelph, Guelph, Ontario, Canada; University of Oslo, NORWAY

## Abstract

**Background:**

Up to 3–5% of adults may be affected by food allergies, while approximately 1% are affected by Celiac disease (CD). Food allergy reactions can be severe and potentially fatal, while CD can result in various symptoms. Restaurant and food service establishment staff have an important role in helping to prevent food allergy and CD risks among affected customers.

**Objectives:**

A systematic review was undertaken to identify, characterize, and synthesize published research on the prevalence of food allergy and CD knowledge, practices, and training among restaurant and food service personnel. The population of interest included any personnel in these settings who prepare, handle, or serve food. Outcomes included the prevalence of food allergy and CD knowledge, practices, and training.

**Methods:**

The review was conducted using standardized methods, including: a comprehensive search strategy; relevance screening of abstracts; characterization of relevant articles; data extraction; and risk of bias assessment. Outcomes were stratified into comparable subgroups and descriptively analyzed to examine prevalence trends across studies. Meta-regression was conducted on selected outcomes to identify possible sources of variability in prevalence estimates across studies.

**Results:**

Thirty-eight relevant studies were identified; most were conducted in the United States (50%) and focused on food allergies (90%). Significant variability was identified across studies for most outcomes. Participants generally had a higher knowledge, self-efficacy, and use of practices related to preparing and serving allergen-free meals compared to food allergy emergency response. Participants’ reported use of various risk prevention and response practices was generally low. Most participants across studies had not received prior food allergy training (median prevalence of 65% across 12 studies).

**Implications:**

Key knowledge and practice gaps were identified that could be targeted by future training programs. Research gaps were also identified, including a need for more experimental studies to evaluate food allergy and CD training interventions.

## Introduction

Food allergies refer to an adverse immune reaction to foods. While it is challenging to determine reliable population-based estimates of the prevalence of food allergies due to the limitations of self-reporting and other methodological issues, previous studies suggest that up to 3–5% of the adult population in North America may be affected by them [1–3]. A food allergy can be triggered by many different types of food; however, the most significant reactions are caused by milk, eggs, peanuts, tree nuts, shellfish, fish, wheat, and soy [1].

Food allergy reactions can range from mild and limited symptoms in the oral cavity to anaphylaxis, which can be fatal if not immediately treated with epinephrine [4,5]. In the United States (US), 63 deaths were identified from 1994–2006 in a voluntary registry of food-induced anaphylaxis fatalities [4,6], and nearly half of these deaths (46%) were caused by food exposure at a restaurant or other food service establishment [7]. Similarly, a study of the Coroner’s database in Ontario, Canada, found that 40 of the 92 identified anaphylaxis deaths from 1986–2011 were food-induced [5], with most cases (24/40) occurring due to exposure outside of the home (e.g. at restaurants, schools and camps) [5].

In addition to food allergies, approximately 1% of the general population in the Western world is affected by Celiac disease (CD) [8,9]. CD is an autoimmune disorder that occurs when an affected individual consumes food containing gluten, a protein found in wheat, barley, rye, and other grains, which results in a variety of intestinal and extraintestinal symptoms [9]. A related illness called nonceliac gluten sensitivity refers to a condition in which symptoms are triggered by gluten ingestion in the absence of CD or wheat allergy related biomarkers, though the extent of the illness and role of gluten in causing symptoms is still unclear [9,10].

Strict avoidance of the causal foods is the primary strategy to prevent food allergies, while maintenance of a lifelong gluten-free diet is the only preventative strategy for CD. Individuals with food allergies and CD face similar challenges when eating out at restaurants and other food establishments due to difficulties in avoiding accidental exposure to food allergens and gluten from cross-contact [11,12]. For example, a previous US study of food allergic individuals in 2007 found that approximately one-third reported experiencing at least one allergic reaction due to eating out at restaurants [13]. Restaurant and food service establishment personnel have an important role in understanding and communicating possible risks to these consumers. In addition, they should be aware of how to rapidly identify and respond to severe anaphylactic reactions due to inadvertent food allergen exposures.

There is a need to better understand the knowledge and practices of restaurant and food service establishment personnel toward the management of consumer food allergies and CD in order to improve consumer experiences when eating out and to prevent accidental allergen exposures. We conducted a systematic review using standardized and structured methods to identify, characterize, and synthesize all available studies in this area, and to examine trends in these outcomes across studies. The review also captured studies reporting on interventions to improve the knowledge and practices of restaurant and food service establishment personnel. Results from this review are useful to inform priority areas for improving the food allergy and CD knowledge and practices of restaurant and food service establishment personnel, including identification of targeted areas for future interventions.

## Materials and methods

### Review question and eligibility criteria

The review was guided by a pre-specified protocol (available from the corresponding author upon request) and was conducted following internationally recommended methodology for systematic reviews [14,15]. This article is reported in accordance with the Preferred Reporting Items for Systematic Reviews and Meta-Analyses (PRISMA) statement [16], and a copy of the PRISMA checklist is available in S1 File. The primary review question was: “What is the prevalence of and variability across studies in the knowledge, practices, and training toward consumer food allergies and CD among restaurant and food service establishment personnel?” Secondary review questions included: “What study-level factors are associated with the variability in the prevalence of these outcomes across studies?”, and “What interventions are effective to improve these outcomes?”

The population of interest was any personnel (e.g. managers, chefs, servers) at restaurants and other food service establishments (e.g. cafeterias, delis) who prepare, handle or serve food to consumers. We included studies of college and university food services, but excluded those investigating personnel at schools and healthcare institutions (e.g. hospitals). Research on food handlers at other stages of the food chain (e.g. processing) were also excluded. The primary outcomes of interest were knowledge, practices, and training, though we also included measures of self-efficacy (e.g. confidence to serve allergen-free food). Eligible sources of evidence included quantitative primary studies published in English, French, or Spanish as journal articles, research reports, dissertations and theses, or conference proceedings.

### Search strategy

A comprehensive search strategy was developed in consultation with a librarian. The search algorithm consisted of a combination of keyword terms extracted from 10 known relevant articles and pre-tested in Scopus. The final algorithm, as implemented in Scopus, was as follows: (restaurant* OR establishment* OR premise* OR cater* OR “food service” OR foodservice OR manager* OR hospitality) AND ((food AND allerg*) OR celiac OR coeliac). The search was implemented on 21 September 2017 in the following bibliographic databases: Scopus, PubMed, CAB Abstracts, Food Safety and Technology Abstracts, PsycINFO, CINAHL, and ProQuest Dissertations and Theses. A complementary search for grey literature documents (e.g. conference proceedings and research reports) was also conducted using a series of 20 simple search strings in Google (e.g. “Food allergy knowledge food handlers”). In addition, we searched the proceedings of the International Association for Food Protection Annual Conference from 2009–2017. The search verification strategy included hand searching the reference lists of all relevant articles, three review articles on the topic, and one book chapter. Additional details on the search are reported in S2 File.

### Relevance screening and confirmation

The titles and abstracts of citations identified during the search were assessed for their relevance to the review using a structured and pre-tested screening form consisting of one yes/no question. Full articles of relevant references were then obtained and confirmed for relevance using a relevance confirmation and characterization form. This form was also used to classify articles according to key characteristics such as publication type and year, study design and location, data collection approach and tools, and details on the target population (e.g. food premise types, personnel characteristics) and outcomes (e.g. knowledge, practices) investigated.

### Risk-of-bias assessment and data extraction

Relevant studies reporting extractable prevalence data for at least one outcome of interest were assessed for risk of bias and outcome data were extracted using two additional forms. Due to a paucity of data, studies reporting on the efficacy of interventions to improve knowledge, practice, or training outcomes were not evaluated at this level. The risk of bias form was developed using assessment criteria modified and adapted from previously developed risk of bias instruments for observational studies [17–19]. Detailed quantitative data on the prevalence of key outcomes (e.g. knowledge, practices) related to food allergies and CD were extracted using a data extraction form. The prevalence data extracted from each study consisted of the numerator and denominator for each outcome. If only a percentage was reported instead of a numerator for any relevant outcomes, the numerator was estimated from the given prevalence value.

### Review management

All references identified through the searches were uploaded to the reference management program RefWorks (ThomsonResearchSoft, Philadelphia, PA). References were de-duplicated and imported into a spreadsheet (Excel 2013, Microsoft Corporation, Redmond, WA) to facilitate the review steps. To ensure rigour in the review process, all steps were conducted by two independent reviewers. The relevance screening form was pre-tested on 30 abstracts before use, while the remaining forms were pre-tested on five articles each. Reviewing proceeded when the kappa agreement was >0.80 for relevance screening, while the other forms were modified as needed to improve clarity and consistent reviewer interpretation. Any reviewer disagreements were resolved by discussion and consensus. A copy of all review forms is available in S2 File.

### Data analysis

We decided not to conduct meta-analysis on prevalence data outcomes extracted from each study because our main interest was in describing the distribution and trends in prevalence across studies rather than calculating an overall (pooled) estimate. In addition, there were substantive differences among studies in the sample populations, their characteristics, and in the measurement instruments used. Therefore, we instead stratified the prevalence data into subgroups that represented similar questions or constructs measured across studies, and summarized the distribution of prevalence estimates within each group descriptively using the median, 25^th^ and 75^th^ percentiles, and range of values across studies. The stratification criteria for this analysis included (in hierarchical order): topic of investigation (food allergies or CD); outcome type (knowledge, practices, or training); and specific question or construct. Study outcomes were only summarized if at least three studies reported the same or similar outcome construct. Subgroups consisting of only one to two studies were excluded at this stage. Forest plots were generated for each outcome subgroup, without an overall meta-analysis estimate, to visualize the variability in prevalence estimates across studies [20]. Self-efficacy outcomes were summarized at the study level instead of using the approach described above because these data were frequently measured on an ordinal scale and response options varied across studies.

Given that large variability in prevalence estimates was expected across studies within most outcome subgroups, we conducted meta-regression analysis to investigate possible study-level factors that might explain these differences [21]. This analysis was only conducted for outcome subgroups comprising at least 10 studies [21]. A series of univariable models were evaluated for each subgroup with the following pre-determined predictor variables: publication year (continuous); document type (journal article vs. other); study country (US vs. other); pre-testing of data collection instruments (yes vs. no); restaurants were investigated as a targeted food premise (yes vs. no); managers or owners were included in the study population (yes vs. no); and the overall risk of bias rating (high/unclear vs. low). Additionally, for practice outcomes, the method of measuring the outcome was also evaluated (observed vs. self-reported). Variables were considered statistically significant if *P*≤0.05. Model variance was estimated using the restricted maximum likelihood method. Residual heterogeneity in the meta-regression models was quantified using *I*^2^, which measures the proportion of variation across studies that is due to study differences rather than sampling error [22]. Forest plots were generated using the *metaprop* command and meta-regression was conducted using the *metareg* command in Stata 14 (StataCorp LP, College Station, USA).

## Results

### Characteristics of relevant studies

**Fig 1:**
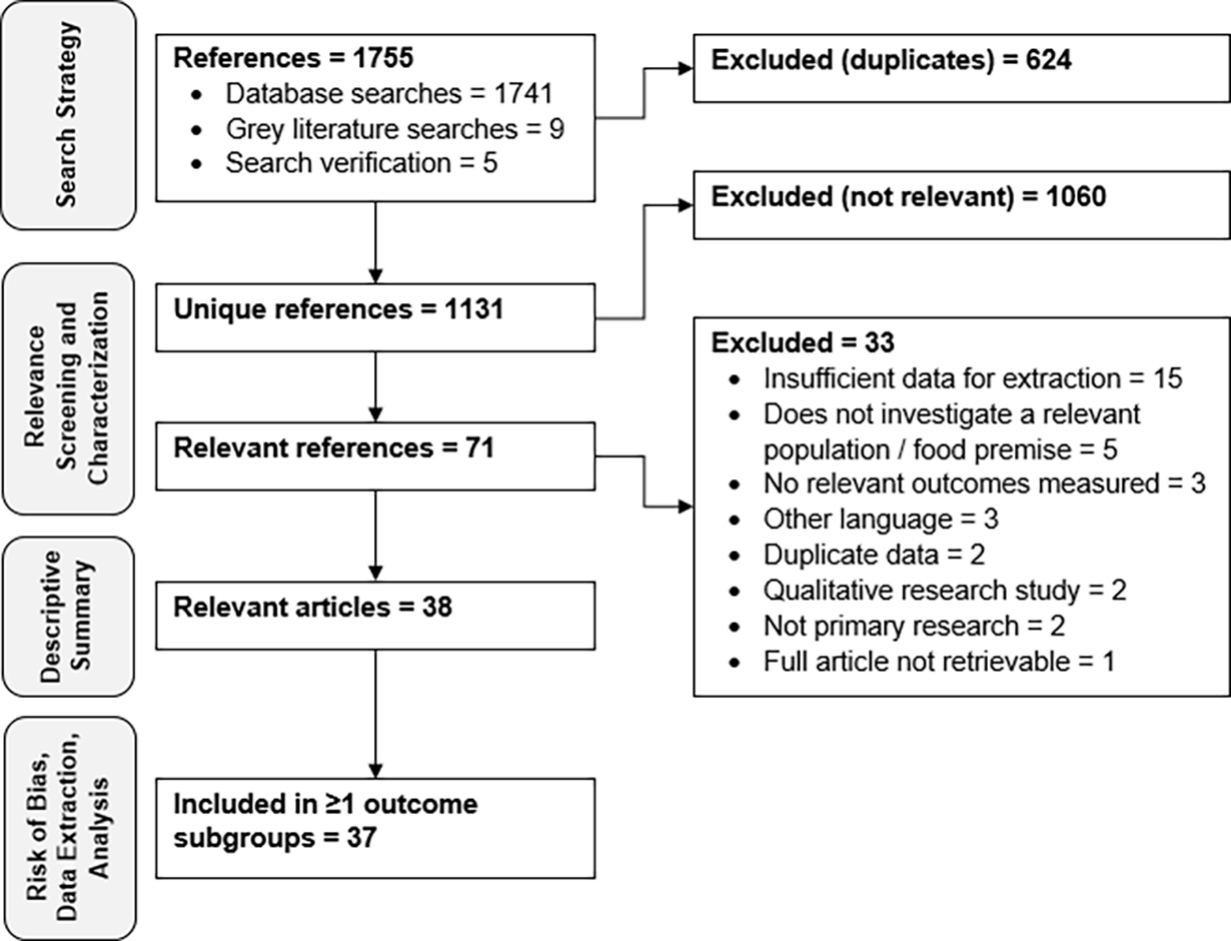
Systematic review flow chart.

A review flow chart is shown in Fig 1. From 1131 unique references screened for relevance, 38 relevant studies were identified (Fig 1). The characteristics of these studies are highlighted in Table 1. Most were published as journal articles (84%), conducted in the US (50%) and United Kingdom (26%), and focused on food allergies (90%) compared to CD (Table 1). The median publication year was 2013 (range 2004 to 2017). Nearly all studies (97%) used a cross-sectional design to evaluate the prevalence of different outcomes, most commonly personnel practices (84%) and knowledge (73%) related to food allergies or CD (Table 1). The most frequently used data collection method was questionnaires (95%), while only 47% of studies reported using a formative research method to develop their data collection instruments, and only 42% pre-tested their instruments (Table 1). Restaurants were the most commonly investigated type of food premise (84%) (Table 1).

**Table 1:** Characteristics of 38 studies that investigated restaurant and food service staff knowledge and practices related to food allergies and CD.

Characteristic	No.	%
Document type:		
Journal article	32	84.2
Conference proceedings paper or abstract	5	13.2
Research report	1	2.6
Study country^a^:		
US	19	50.0
United Kingdom	10	26.3
Malaysia	2	5.3
New Zealand	2	5.3
Other^b^	6	15.8
Study topic of investigation^a^:		
Food allergies	34	89.5
Celiac disease (CD)	9	23.7
Study focus of investigation^a^:		
Prevalence of outcomes	37	97.4
Efficacy of intervention	2	5.3
Study design^a^:		
Cross-sectional	37	97.4
Experimental	2	5.3
Data collection methods^a^:		
Questionnaire:	36	94.7
In-person	17	44.7
Postal	9	23.7
Web-based	8	21.1
Telephone	4	10.5
Not specified	3	7.9
Participant observation	5	13.2
Food sampling for allergens	3	7.9
Formative research methods used to inform development of data collection instruments^a^:		
Previous surveys / research	18	47.4
Allergy training resources	3	7.9
Expert panels	2	5.3
Participant interviews	2	5.3
Food premise observations	1	2.6
None reported	20	52.6
Theories of behaviour change used to inform development of data collection instruments:		
Yes	0	0.0
No	38	100.0
Methods used for pre-testing of data collection instruments^a^:		
Pilot study	10	26.3
Expert review	10	26.3
Not specified	4	10.5
None reported	22	57.9
Method of participant recruitment specified:		
Yes	28	73.7
No	10	26.3
Study response rate reported:		
Yes	20	52.6
No	18	47.4
Types of food premises investigated^a^:		
Restaurants	32	84.2
Cafes, pubs, and bars	5	13.2
Colleges and universities	5	13.2
Delis	3	7.9
Lodging facilities (e.g. hotels)	2	5.3
Caterers	2	5.3
Bakeries	1	2.6
Not specified	4	10.5
Types of staff investigated^a^:		
Managers, supervisors, and owners	23	60.5
Chefs and other food workers (e.g. cooks)	23	60.5
Servers	16	42.1
Non-food handlers (e.g. hosts, dieticians)	6	15.8
Food premise observations	3	7.9
Hospitality management / culinary students	2	5.3
Not specified	9	23.7
Study targeted ethnic-operated food premises:		
Yes	2	5.3
No	36	94.7
Outcome categories of interest measured^a^^,^^c^:		
Practices and behaviours	31	83.8
Knowledge	27	73.0
Training experience and policies	23	62.2
Self-efficacy	10	27.0

^a^ Multiple selections were possible for these questions, and percentages may not add to 100%.

^b^ Other countries were investigated in one study each, and included: Argentina; Brazil; Ireland; the Netherlands; Spain; and Turkey.

^c^ Percentages for this question were tabulated out of the number of studies investigating prevalence outcomes (n = 37).

The summary risk of bias ratings of studies that provided sufficient data for one or more relevant prevalence outcomes (n = 37) are shown in Table 2. Most studies (65%) reported at least one outcome that was rated as unclear risk of bias, mostly due to uncertainty about the representativeness of the study population and a lack of clarity about the validity and reliability of data collection instruments (Table 2). Full citation details, extracted characteristics, and individual risk of bias ratings for each relevant study are reported in S1 Dataset. In addition, a copy of the study-level prevalence data for each outcome, including forest plots not reported in this manuscript, are also reported in S2 Dataset and S3 File.

**Table 2:** Risk of bias assessment summary for 37 studies that investigated restaurant and food service staff knowledge and practices related to food allergies and CD.

Risk of bias criteria	No. of unique outcome assessments^a^	No. (%)^a^^,^^b^		
		Low risk	Unclear risk	High risk
Study participants likely to be representative of the target population	38	12 (32%)	24 (65%)	2 (5%)
Use of valid and reliable instruments to measure outcomes	37	12 (32%)	25 (68%)	0 (0%)
Losses to follow-up (attrition) and/or exclusions from analysis reported	37	32 (86%)	5 (14%)	0 (0%)
Author reporting of all intended outcomes	37	29 (78%)	7 (19%)	1 (3%)
Other potential biases	38	37 (100%)	1 (3%)	0 (0%)
Overall risk of bias rating	39	12 (32%)	24 (65%)	3 (8%)

^a^ All percentages were calculated using the total number of relevant studies (n = 37) as the denominator, so will add to more than 100% for criteria where some studies reported multiple outcomes that had different risks of bias.

^b^ Risk of bias rating definitions: Low risk = plausible bias unlikely to significantly alter the results; unclear risk = plausible bias that raises some doubt about the results; high risk = plausible bias that considerably weakens confidence in the results [14].

### Food allergy prevalence outcomes

We identified 30 unique knowledge outcome subgroups, eight practice outcome subgroups, and three training subgroups. A descriptive summary of each of these outcomes is reported in S1 Table. For most outcome subgroups, substantial variability in prevalence was noted across studies (S1 Table).

**Fig 2:**
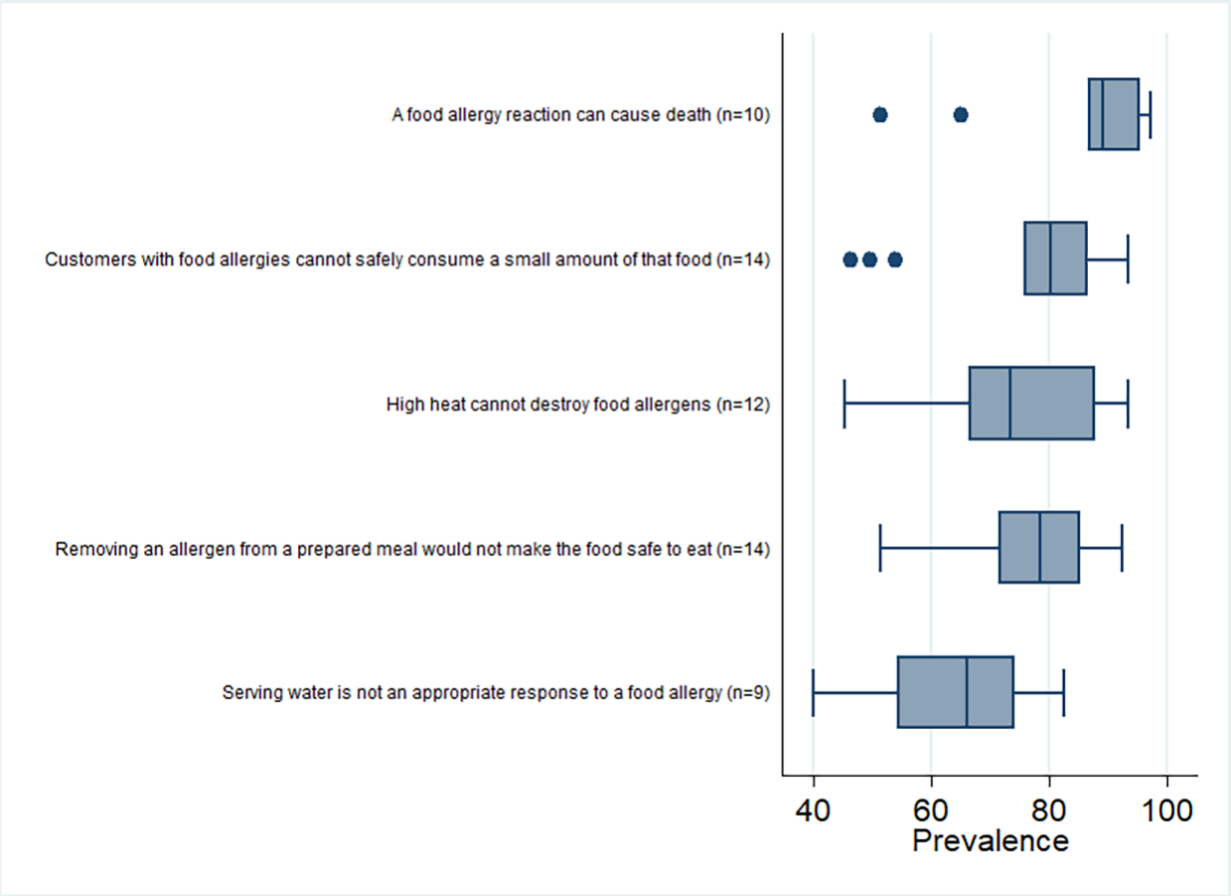
Box plot of the across-study prevalence of correct responses to five knowledge questions about food allergies.

A summary of the five most frequently investigated knowledge outcomes is shown in the box plot in Fig 2. High levels of knowledge were noted across studies for the fatal consequences of food allergies (median prevalence = 89%; n = 10 studies), that customers cannot eat even small amounts of foods containing allergens (median = 80%; n = 14), and that removing an allergen from a prepared meal would not make the meal safe to eat (median = 79%; n = 14). In contrast, a consistently lower level of knowledge (median prevalence = 66%; n = 9) was noted for whether serving water is an appropriate response strategy to someone experiencing a food allergic reaction.

Across studies, most participants were correctly able to identify the major food allergens from a given checklist (S1 Table). The allergens identified by the fewest participants across studies (n = 5 each) included wheat, fish, and soy (median prevalence values of 72%, 60%, and 54%, respectively; S1 Table). Most participants were correctly able to identify possible food allergy symptoms from a given checklist in studies that asked this question (S1 Table). However, surprisingly, anaphylaxis was only identified by a median prevalence of 72% of participants in four studies (range 66–75%), which was less than all other listed symptoms except for vomiting (S1 Table).

**Fig 3:**
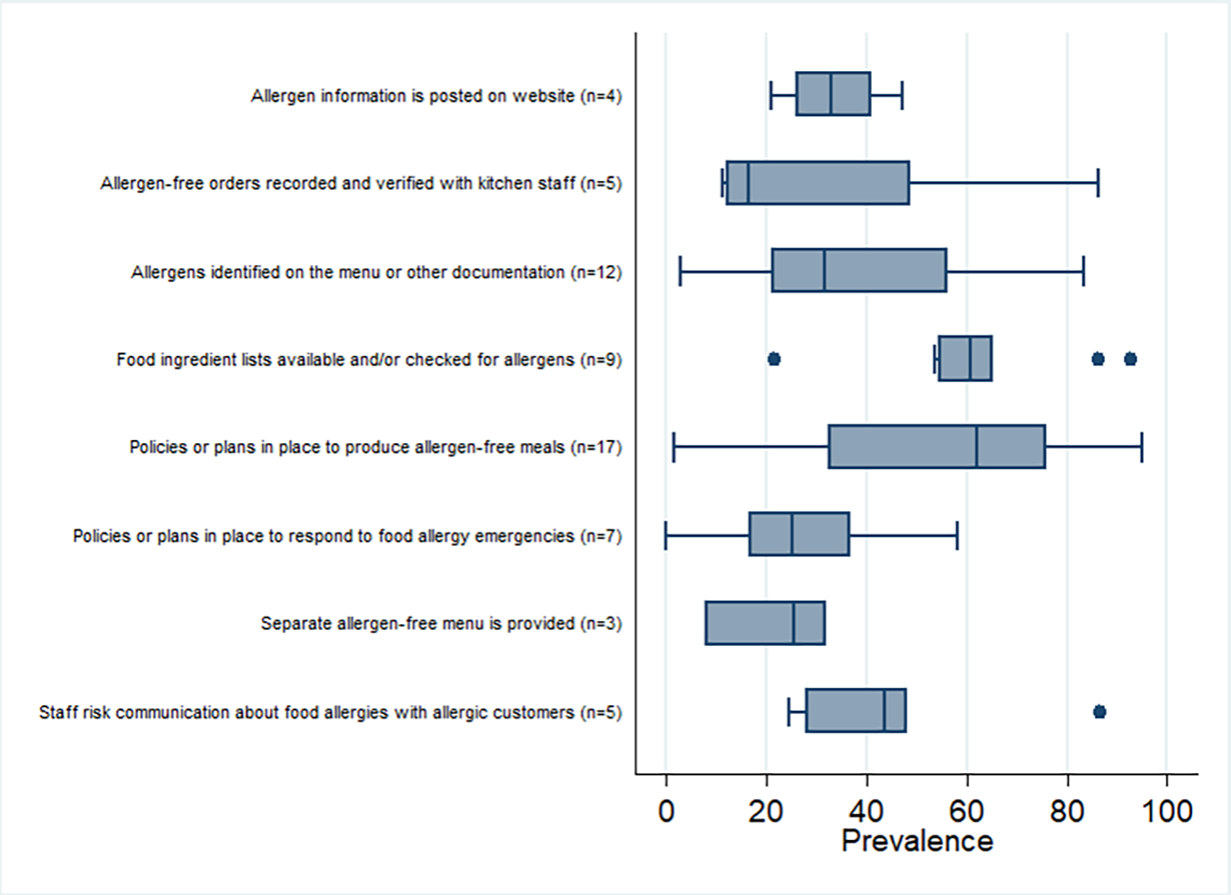
Box plot of the across-study prevalence of food allergy practices and behaviours.

**Fig 4:**
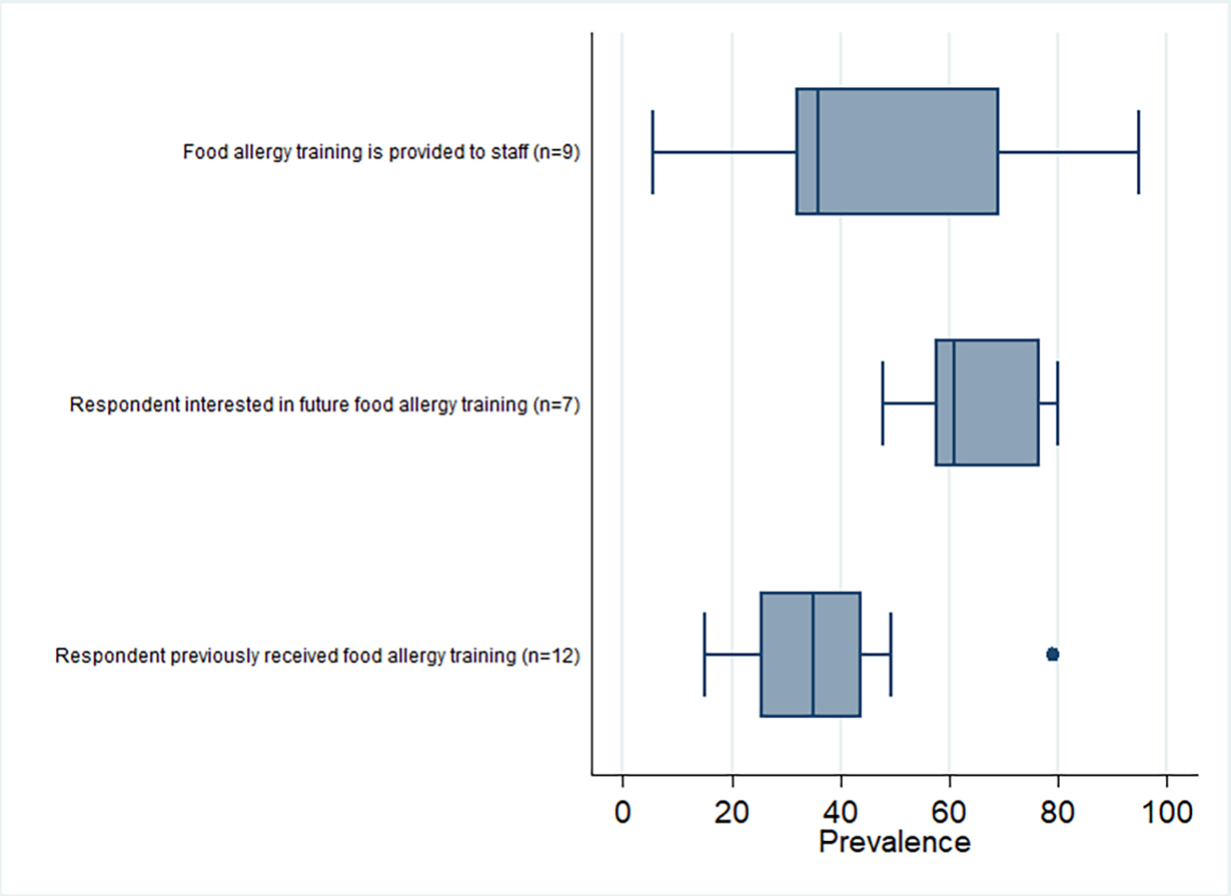
Box plot of the across-study prevalence of food allergy training outcomes.

**Fig 5:**
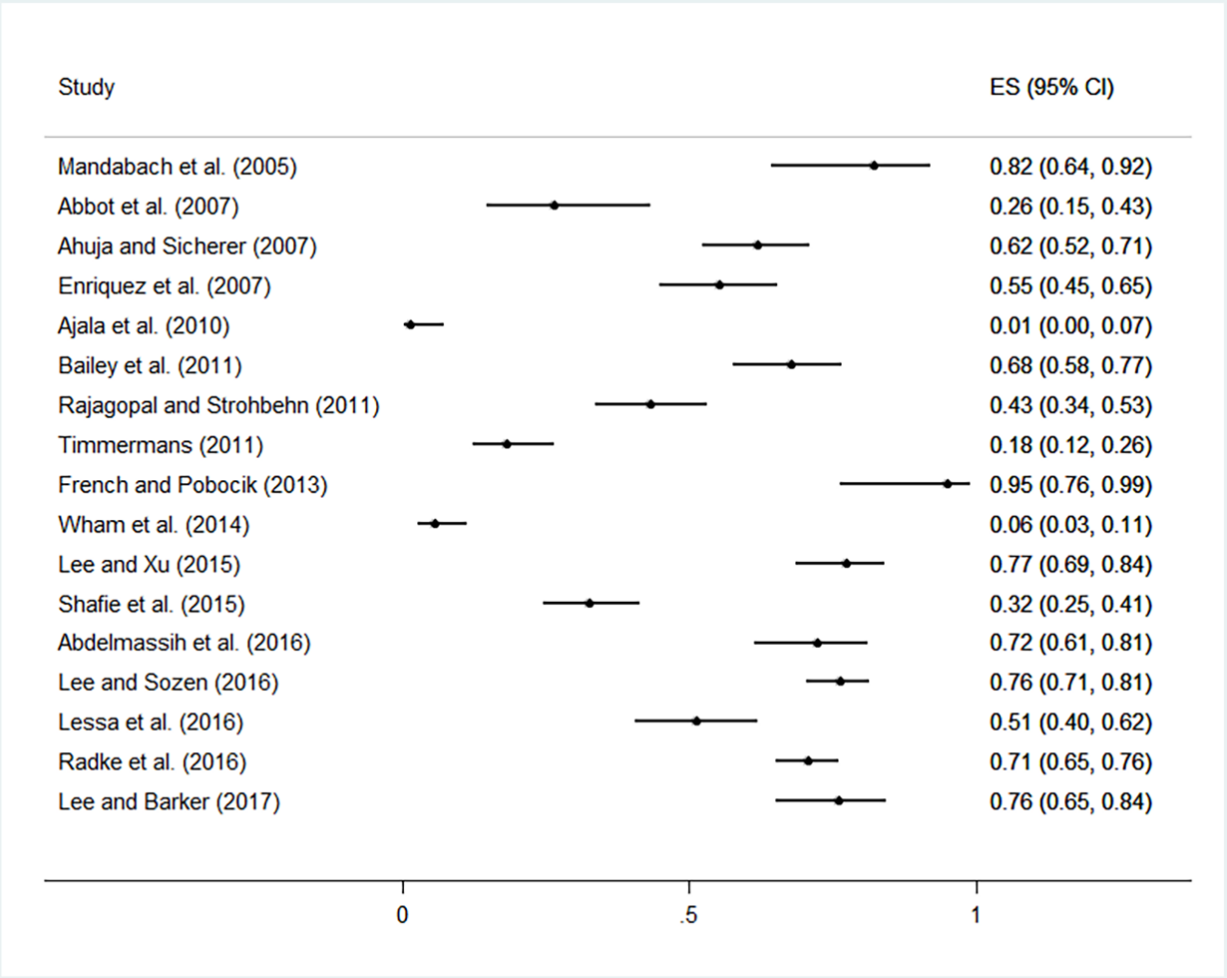
Forest plot of the prevalence of having policies or plans in place to produce allergen-free food.

**Fig 6:**
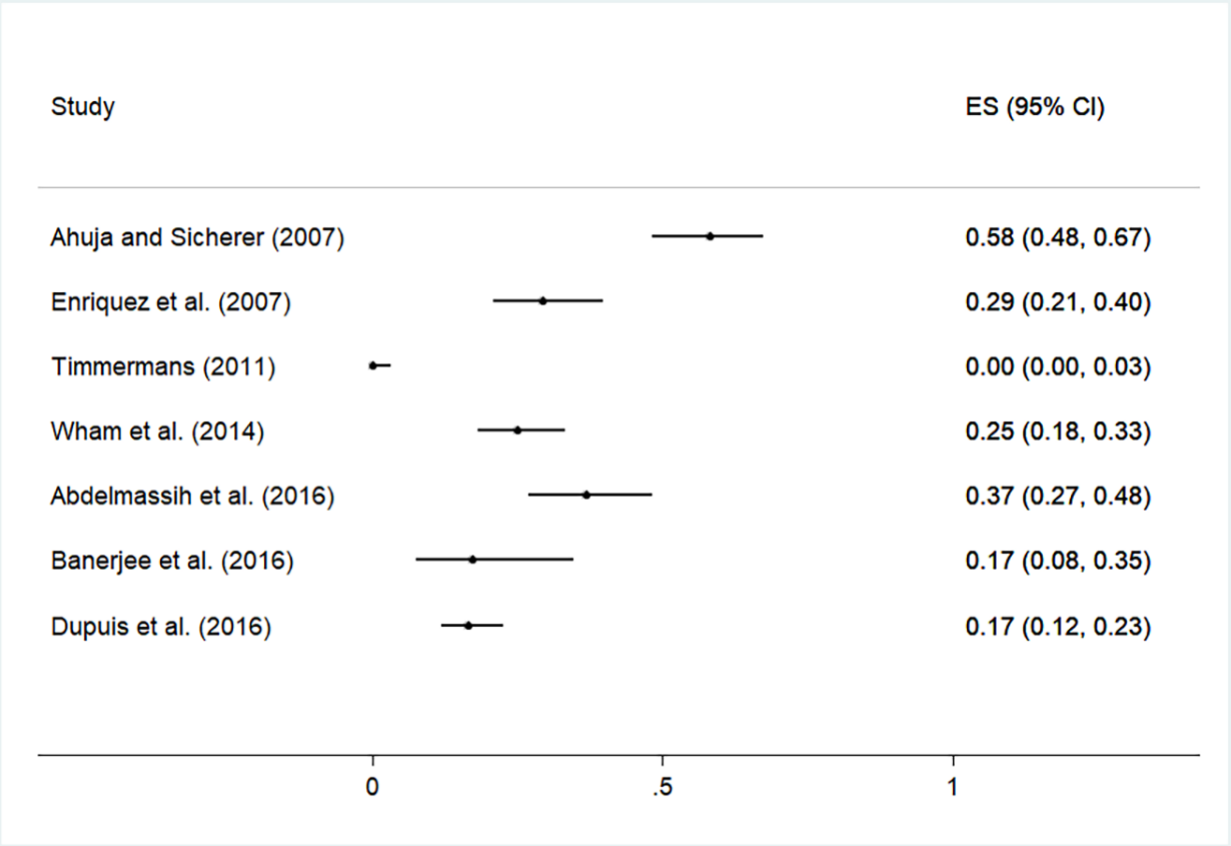
Forest plot of the prevalence of having policies or plans in place to respond to food allergy emergencies.

**Fig 7:**
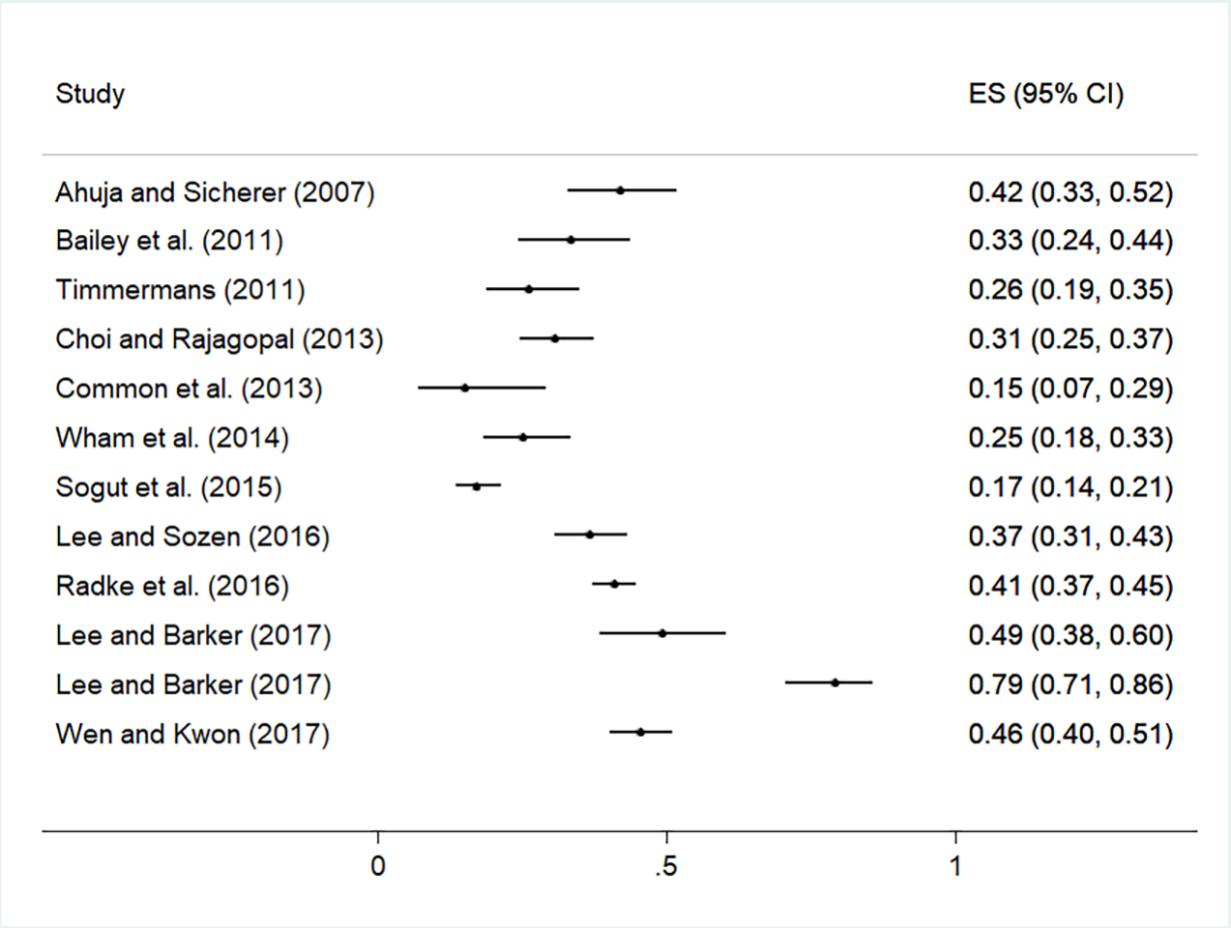
Forest plot of the prevalence of respondents indicating that they have received prior food allergy training.

The practice and training outcomes are highlighted in Fig 3 and Fig 4, respectively. The practices and behaviours most frequently reported by participants across studies included having a policy or plan to produce allergen-free meals and having food ingredient lists available or checked for food allergens as necessary (median prevalence values of 62% and 61% in 17 and 9 studies, respectively). The median prevalence of all other practices and behaviours was <50% (S1 Table and Fig 3), including having a policy or plan in place to respond to food allergy emergencies (median prevalence = 25%, n = 7). Forest plots are shown in Fig 5 and Fig 6 to visualize the across-study prevalence of having policies or plans in place to produce allergen-free meals and respond to emergencies, respectively. While most participants across studies indicated that they were interested in food allergy training (median prevalence = 61%; n = 7), most had not received prior training and indicated that training was not provided in their establishment (median = 35% and 36% in 12 and 9 studies, respectively). A forest plot of the studies reporting on prior food allergy training among participants is shown in Fig 7.

A summary of self-efficacy outcomes related to food allergy is shown in S2 Table. The results suggest that most participants were confident in their ability to prepare or serve allergen-free meals compared to responding to food allergy emergencies (S2 Table). Only four outcome subgroups were identified related to CD (S1 Table). Participants’ self-reported knowledge of CD and gluten sensitivity was generally high for most studies (median prevalence of 77% and 88% in 7 and 3 studies, respectively). While the median prevalence of reporting that gluten-free foods are available at the food establishment was high (84%; n = 4 studies), studies reported a lower median prevalence (50%; n = 4) of participants who indicated that the availability of gluten-free foods is indicated on the menu or other documentation (S1 Table).

### Meta-regression results

Meta-regression was possible for seven outcome subgroups, including four knowledge outcomes, two practice outcomes, and one training outcome (Table 3). The results are shown in Table 3. Studies conducted in the US vs. other countries were more likely to report a higher prevalence of participant knowledge of two food allergy questions, participants reporting that policies or plans are in place to produce allergen-free meals, and participants reporting that they had previously received food allergy training (Table 3). Studies that were published as journal articles compared to other sources were more likely to report participants correctly identifying that high heat cannot destroy food allergens (Table 3). No significant predictors were identified for the fourth knowledge outcome: that removing an allergen from a prepared meal would not make it safe to eat. Three significant predictors were identified for the practice of identifying allergens on the menu or other documentation (Table 3). Studies published in more recent years, studies that did not include restaurants as a targeted food premise, and studies that measured this outcome via self-reporting (vs. in-person observations) were all more likely to report a higher participant prevalence of this practice (Table 3).

**Table 3:** Summary of significant predictors of across study heterogeneity as identified in meta-regression analyses of selected outcome subgroups.

Meta-regression subgroup / predictor^a^	No. of studies	*Beta* (95% CI)	*P* value	*I*^2^	Adj. *R*^2^
**Knowledge = a food allergy reaction can cause death**					
Study conducted in the US (yes vs. no)	10	0.29 (0.17, 0.42)	0.001	8.2%	100%
**Knowledge = customers with food allergies cannot safely consume a small amount of that food**					
Study conducted in the US (yes vs. no)	14	0.17 (0.00, 0.33)	0.050	64.2%	34.7%
**Knowledge = high heat cannot destroy food allergens**					
Document type (journal article vs. other)	12	0.31 (0.00, 0.63)	0.049	56.0%	36.0%
**Practice = allergens are identified on the menu or other documentation**					
Publication year (continuous)	12	0.03 (0.01, 0.06)	0.021	66.2%	52.3%
Food premises included restaurants (yes vs. no)	12	-0.44 (-0.84, -0.04)	0.033	70.2%	37.2%
Method of measuring the practice (observed vs. self-reported)	12	-0.30 (-0.55, -0.04)	0.027	68.3%	43.4%
**Practice = policies or plans are in place to produce allergen-free meals**					
Study conducted in the US (yes vs. no)	17	0.37 (0.15, 0.59)	0.003	70.3%	54.9%
**Training = respondent has previously received food allergy training**					
Study conducted in the US (yes vs. no)	12	0.22 (0.05, 0.38)	0.016	53.8%	58.6%

Adj. = adjusted; CI = confidence interval.

^a^ A fourth knowledge outcome was also investigated, “removing an allergen from a prepared meal would not make it safe to eat”, but no significant predictors were identified.

### Interventions studies

Only two studies were identified that evaluated the efficacy of an intervention to improve personnel knowledge or practices related to food allergies [23,24]. One study in the United Kingdom evaluated the impact of a one-hour lecture to improve restaurant personnel knowledge about food allergies in an uncontrolled before-and-after (i.e. pre-post) design [23]. The authors found that the intervention improved participants’ overall knowledge scores and ability to identify common food allergens [23]. Another study in the US conducted a randomized controlled trial (RCT) to evaluate the impact of a “food allergy alert card”, comparing a version with a photograph and descriptive text of a hypothetical child customer with food allergies to another version with the text description of the child but no photograph [24]. The study did not find any substantive differences between the two groups for a number of measured outcomes, including attitudes, self-efficacy, practices, and knowledge [24].

## Discussion

We used a structured and transparent systematic review approach to identify, characterize, and synthesize all available global research on the knowledge, practices, and training related to food allergies and CD among restaurant and food service personnel. Nearly all of the relevant studies were conducted in either the US (50%) or United Kingdom (26%), indicating a lack of globally representative research on this topic. Many studies based their questionnaire on earlier published surveys in this field [25,26]; however, few studies pre-tested their questionnaires and none were explicitly guided by a theory of behaviour change. A lack of information on validity and reliability testing of questionnaires and the representativeness of study populations contributed to unclear risk of bias ratings for some studies. For example, some studies evaluated convenience samples of food service personnel that might not be representative of the larger food service populations in those settings. These limitations should be considered when interpreting the results of this review; we encourage future authors in this area to consider these issues during study design and to follow internationally adopted reporting guidelines [27].

We found that the food allergy knowledge, practices, and training among participants were highly variable across studies (S1 Table). Many knowledge gaps were identified, suggesting a need for increased education and training of restaurant and food service personnel. A particular concern is that many studies found lower levels of participant knowledge, practices, and self-efficacy of responding to food allergy emergencies compared to producing and serving allergen-free meals. In addition, fewer participants across studies identified anaphylaxis as a possible food allergy reaction compared to several other symptoms, which could be due to a lack of familiarity with this medical term. Both prevention of and response to food allergy incidents are important to protect food allergic individuals and to mitigate the risk of potentially fatal anaphylaxis. However, previous research has found that food allergy training programs for restaurant and food service personnel tend to focus more on risk communication with customers and avoiding cross contact rather than recognizing symptoms of and responding to a potential reaction [28–30]. Given the need for immediate medical attention and prompt administration of epinephrine during an allergic reaction [5,7], information about responding to food allergy emergencies should receive enhanced focus in training initiatives.

Many studies found that allergens were not identified on the menu or other documentation (e.g. signage), nor on the website, of participating food premises, and gaps were found in the use of several other recommended practices (e.g. risk communication with customers). These results are concerning because food allergic consumers often rely on written information about food allergens when eating out to help them avoid potential exposure to food allergens [12,31]. In addition, previous studies have reported that food allergic customers do not have a high degree of trust in restaurant and food service personnel and face numerous challenges when communicating with them about food allergies (e.g. miscommunication, fear of social embarrassment) [12,31,32]. Enhanced efforts are needed by restaurant and food service personnel to build trust with food allergic customers and to provide them with accurate and reliable information about food allergen risks.

Many jurisdictions require food safety training and certification for food handlers working in the retail and food service industry. While these courses provide basic information about food allergies, more comprehensive and specialized training in food allergies has been recommended [25,28,33,34]. For example, the US Food Code states that all persons in charge of a food premise should ensure that their employees are properly trained in food allergy awareness [35]. We found that many restaurant and food service personnel across studies were interested in receiving food allergy training; however, fewer participants reported that they had received previous training and that food allergy training is provided in their establishment (Fig 4). Future research is necessary to investigate how to improve food allergy training opportunities for restaurant and food service personnel.

We were able to identify a number of predictors of the variability in prevalence estimates across studies through meta-regression analyses (Table 3). Studies that were conducted in the US (vs. other countries) were more likely to report a higher prevalence of participant knowledge of two food allergy questions, participant indication that policies or plans are in place to produce allergen-free meals, and that participants had previously received food allergy training. This could be due to increased attention toward this issue, as referenced in the US Food Code [35], as well as the presence of legislation in some states that requires food allergy training and other education strategies [36]. Studies published as journal articles reported a higher prevalence of participant knowledge that high heat cannot destroy food allergens compared to one conference proceeding article in the same subgroup. This finding corresponds with previous research which found that studies published only in conference proceedings tend to report more conservative effects compared to those in journal articles [37].

Three predictors were identified for the practice of reporting that allergens are identified on the menu or other documentation. The significance of publication year suggests that more restaurants and food service establishments have adopted this practice in recent years. Studies that included restaurants as a targeted food premise reported a lower prevalence of participant use of this practice compared to other premises, all of which included college and university food services. College and university food services may be more likely to identify allergens due to the need to accommodate food-allergic students with meal plans, and in the US, they may be influenced by previous legal action in 2013 against a university for non-compliance with the Americans with Disabilities Act (ADA) due to insufficient accommodation of students with special dietary needs [38]. Studies that measured the identification of food allergens on menus and other documentation through in-person observations (compared to participant self-reporting) reported a lower use of this practice. This finding is in agreement with previous research that has found that restaurant and food service staff tend to overestimate their safe food handling practices on self-report surveys [39–42].

We identified only two studies that evaluated the efficacy of interventions to improve restaurant and food service personnel knowledge or behaviours related to food allergies [23,24], indicating that further research is necessary in this area. Several online training courses in food allergies have been developed for restaurant and food service personnel (e.g. Allergen Training Basics in Canada, ServSafe Allergens in the US) [43–45]. However, we were not able to identify any published evaluations of these or other similar programs. Some of the studies captured in this review found that previous food allergen training was associated with higher levels of food allergen knowledge [29,30,46], attitudes [47], and practices [30,48] among participants, supporting the importance of training to improve these outcomes. However, more experimental studies, ideally using RCT designs, are needed in this area.

We identified comparatively less research focused on knowledge and practices related to CD (S1 Table). Although there was wide variability in these outcomes, awareness was generally high and has improved in recent years [49–52], while gaps were identified in the reporting of gluten-free food availability on menus or other documentation. As with food allergic customers, individuals with CD often rely on written information about the gluten status of meals to inform their purchasing and dining decisions, and they also face various challenges (e.g. social frustration and isolation) when eating out at restaurants [31,53,54]. More research is needed to investigate the CD training status of restaurant and food service personnel, including how to improve their risk prevention and communication practices.

There are several limitations of this review. Publication bias is a potential concern, in that smaller studies with systematically different results might exist on this topic but may not have been published [55]. This may be particularly a concern for less recently published studies [56]. We did not implement formal statistical tests to investigate this bias because the tests require many assumptions to produce reliable results and may not be suitable for prevalence outcomes from observational studies [55,57]. However, the meta-regression finding that conference proceedings tended to report a lower prevalence for one knowledge outcome compared to journal articles suggests possible publication bias for this topic, and also highlights the importance of including grey literature in systematic reviews. A related concern is the possibility of missing some relevant articles due to a lack of search term sensitivity or database coverage. In order to mitigate these potential biases, we pre-tested search terms, searched multiple databases, searched for grey literature (including hand-searching one relevant conference proceedings in the topic area), and implemented a verification strategy. However, despite these efforts, it is still possible that we missed some relevant articles or grey literature.

Studies examining food service personnel in schools and healthcare institutions were excluded from this review; the food allergy knowledge, practices, and training in these settings may differ from those reported in this review. We grouped the prevalence data from each study into comparable subgroups that represented similar outcome constructs. While a descriptive analysis of the distribution of prevalence estimates across studies within each subgroup has highlighted trends in these outcomes, the study populations, outcome measurement methods, and other factors (e.g. response rates) differed substantially across studies and likely contributed to the observed variability in estimates for most outcomes. The meta-regression analysis was useful to provide insights into possible factors associated with this variability in prevalence across studies. However, this method has some key limitations, including low power to detect true predictors of heterogeneity (given that the evaluated models contained only 10–17 studies), as well as the possibility of identifying false positive findings (which we attempted to mitigate by limiting analysis to a pre-specified list of possible predictors) [21].

## Conclusions

This systematic review has synthesized the global research on restaurant and food service establishment personnel knowledge, practices, and training related to food allergies and CD. Key research gaps were identified related to the evaluation of interventions to improve food allergy knowledge and practices in these settings, and the extent of food service personnel knowledge and practices toward CD. The results suggest a need for increased training opportunities for restaurant and food service establishment personnel in food allergy prevention and response (including identification of anaphylaxis), as well as CD and the gluten free diet. In addition, enhanced identification of food allergens (e.g. on menus) and risk communication practices in restaurants and other food service settings would help to support food allergic individuals to make informed decisions when eating out and to avoid possible allergen exposures.

## Supporting information

S1 File PRISMA checklist. (DOCX)

S2 File Review forms and search details. (DOCX)

S3 File Prevalence outcome forest plots. (DOCX)

S1 Dataset Study characteristics and citation information. (XLSX)

S2 Dataset Study level prevalence outcome data. (XLSX)

S1 Table Descriptive summary of knowledge, practice, and training outcomes. (DOCX)

S2 Table Descriptive summary of self-efficacy outcomes. (DOCX)
