# Supplementary material for: A systematic review and meta-regression of the knowledge, practices, and training of restaurant and food service personnel toward food allergies and Celiac disease
Source: PLoS One. 2018 Sep 4;13(9):e0203496. doi: 10.1371/journal.pone.0203496 (PMC6122805; doi:10.1371/journal.pone.0203496)
Supplement: S2 File — (DOCX) [file pone.0203496.s002.docx]

S2 File: Systematic Review Forms and Search Details

**Relevance Screening Form**

| **Question** | **Options** | **Definitions/additional notes** |
| --- | --- | --- |
| 1. Does the citation describe primary research that investigates knowledge, attitudes, or behaviours of restaurant or food service staff toward food allergies or food intolerances? | - Yes - No | *Food allergies / food intolerances*  Allergies include any immune response (including anaphylaxis) to foods such as milk, eggs, peanuts, tree nuts, shellfish, fish, wheat, and soy. Intolerances of interest primarily include Celiac/coeliac disease and other gluten sensitivities, but research on other food intolerances (e.g. lactose) should also be included.  *Restaurant / food service staff:*  Includes cooks, chefs, servers, managers, caterers, butchers, and other food employees who handle, serve and/or prepare food at retail / food service. **Exclude studies on consumers who prepare food at home, as well as those investigating staff at schools and institutions (e.g. hospitals). Also exclude studies conducting at the processing/manufacturing level.**  *Knowledge, attitudes, or behaviours:*  Includes studies investigating these as well as any other social and psychological factors that might be related to food allergy/intolerance management at restaurants and food safety. Examples: self-efficacy; subjective norms/social influences; motivators; facilitators; barriers; challenges; past behaviours/habits; social environment constraints (e.g. lack of time, training or resources).  *Include*   - All primary research study designs, publication dates, and types, including grey literature (e.g. theses, dissertations, government/research reports) - Studies where you “can’t tell” the relevance and suspect it could be relevant   *Exclude*   - Research related to ONLY non-allergen hazards (e.g. microbial food safety). |

**Article Characterization and Charting Form**

| **Question / Excel column code** | **Options** | **Comments** |
| --- | --- | --- |
| 1) Is this a primary quantitative research study that investigates the knowledge, attitudes, or behaviours of restaurant or food service staff toward food allergies or food intolerances, published in English, French or Spanish?  Q1_relevant | Yes  No, specify reason(s) for exclusion:   - No relevant data for extraction and synthesis - Not relevant to review question: _________ - Other language: _________ - Qualitative research - Not primary research - Other:____________   If “no” is selected, submit form without proceeding further. | *Food allergies / food intolerances*  Allergies include any immune response (including anaphylaxis) to foods such as milk, eggs, peanuts, tree nuts, shellfish, fish, wheat, and soy. Intolerances of interest primarily include Celiac/coeliac disease and other gluten sensitivities, but research on other food intolerances (e.g. lactose) should also be included.  *Restaurant / food service staff:*  Includes cooks, chefs, servers, managers, caterers, butchers, and other food employees who handle, serve and/or prepare food at retail / food service. Exclude studies on consumers who prepare food at home, as well as those investigating staff at schools and healthcare institutions (e.g. hospitals). Also exclude studies conducting at the processing/manufacturing level.  *Knowledge, attitudes, or behaviours:*  Includes studies investigating these as well as any other social and psychological factors that might be related to food allergy/intolerance management at restaurants and food safety. Examples: self-efficacy; subjective norms/social influences; motivators; facilitators; barriers; challenges; past behaviours/habits; social environment constraints (e.g. lack of time, training or resources).  **Quantitative primary research:** Investigator(s) collected samples or data themselves for analysis- usually a numerical summarization (e.g. cross-sectional studies, case-control studies).  **Qualitative research:** Aimed at understanding social phenomena, exploring issues, and answering questions of “why” and “how” as opposed to numerical summarization of results. Not usually generalizable to a whole population. Examples: focus groups, interviews. |
| 2) What is the publication year of this article?  Q2_year | _______ |  |
| 3) What type of document is this article?  Q3_document | Journal article  Thesis  Conference paper/abstract  Government or research report  Other, please specify:_____ |  |
| 4) What is the article language?  Q4_document | English  French  Spanish | **Exclude** studies in languages other than English, French, or Spanish |
| 5) Where was the study conducted?  *(Check all that apply)*  Q5a_region  Q5b_country | North America:   - Canada - USA - Mexico   Europe:______________  Australasia:_____  Central and South America/ Caribbean:________  Asia:_____________________  Africa:____________________  Not stated | **Central-South America/Caribbean:** [Please specify country only and use full name e.g. Columbia; do not include region etc.]  **Europe:** includes, Belarus, Latvia, Ukraine, Estonia, Cyprus & west (inc. Iceland and Greenland) [please specify country only and use full name].  **Asia:** Russia, Turkey, middle eastern countries and east  **Australasia** is limited to Australia, New Guinea, New Zealand, New Caledonia, and neighbouring islands |
| 6) When was the study conducted?  Q6_date | ___________  Not reported | Please specify year/month to year/month if available (do not extract days) [follow format example: 2000/06-2000/08] |
| 7) What type of allergies/ intolerances did the study focus on?  Q7a_focusgeneral  Q7b_focusspecific  Q7c_focusceliac  Q7d_focusgluten  Q7e_focusother | General food allergies  Specific type of food allergy, specify:_______  Celiac disease  Non-celiac gluten sensitivities  Other food intolerance, specify:____ | **Specific type of food allergy =** only answer if study only focused on one or more major allergens, e.g. milk, eggs, peanuts, tree nuts, shellfish, fish, wheat, soy. |
| 8) What is the study design?  *(Check all that apply)*  Q8a_observational  Q8b_observationalspecific  Q8c_experimental  Q8d_experimentalspecific  Q8e_qualitative  Q8f_otherdesign | Observational study:   - Cross-sectional - Longitudinal - Cohort - Case-control - Other:______________   Experimental study:   - Controlled trial - Uncontrolled before-and-after study - Interrupted time series (ITS) - Other:_______________   Qualitative study, please specify:_______  Other, please specify:_______ | **If more than one design, report ONLY study design(s) relevant to the research question.**  **Observational study:** Assignment of subjects into a treated group versus a control group is natural (outside the control of the investigator).   - **Cross-sectional:** Examines the relationship of a risk factor/predictor and outcome at a point in time on sample of the target population. - **Longitudinal:** Examines the relationship of a risk factor/predictor and outcome at multiple points in time on sample of the target population. - **Cohort study**: is a study in which one or more groups of individuals with differing exposures to a suspected risk factor/predictor are observed and followed through time for occurrence of an outcome. - **Case-control study**: compares exposure to the risk factor/predictor in subjects who have an outcome ('cases') with those who don’t have the outcome, but are otherwise similar ('controls') and drawn from the same sampling frame.   **Experimental study:** Each subject is assigned to a treated group or a control group before the start of the treatment   - **Controlled trial:** an experimental study in which people are allocated to intervention/comparison groups and evaluated for outcomes. Randomized (RCT) if authors specifically indicate random allocation of treatment/control. - **Uncontrolled before-and-after study:** observations are made on a population before and after receiving an intervention. - **Interrupted-time-series (ITS) study:** A study that uses observations at multiple time points before and after an intervention (the ‘interruption’). Differs from uncontrolled before-after study in that multiple measurements (at least 3) are made before AND after the intervention. |
| 9) How were the data collected?  Q9a_questionnaire  Q9b_questionnairespecific  Q9c_observation  Q9d_observationspecific  Q9e_focusgroups  Q9f_interviews  Q9g_othercollection  Q9h_notspecified | Questionnaire:   - In-person - Telephone - Postal - Web-based   Participant observation:   - Direct observation (in person) - Video cameras - Other, specify:___   Focus groups  Interviews  Other, please specify ______  Not specified |  |
| 10) Were the data collected on more than one occasion (e.g. multiple surveys implemented at different time points)?  Q10a_timepoints  Q10ba_timepointsnumber | Yes, specify number of occasions:___  No | E.g. for longitudinal or intervention (before-and-after) studies |
| 11) What was the overall theme of investigation?  Q11a_themeprevalence  Q11b_themeassociation  Q11c_themeintervention  Q11d_themeother  Q11e_na_qual | Prevalence of knowledge, attitudes, or behaviours  Factors associated with knowledge, attitudes, or behaviours  Intervention to improve knowledge, attitudes, or behaviours  Other, please specify ______  N/a - qualitative study |  |
| 12) Was the study design informed by any theories of behaviour change?  Q12a_theory  Q12b_theoryspecific | Yes, specify:   - Theory of Planned Behaviour/Reasoned Action - Health Belief Model - Stages of Change Theory/Transtheoretical Model - Other:_______________   No / not specified | For prevalence/association studies, this question primarily applies to the development of the data collection instruments (i.e. questionnaires). For intervention studies, it primarily applies to the development of the intervention.  **Select any of the options ONLY IF AUTHORS EXPLICITLY INDICATED the theory was used.** |
| 13) Were the data collection instruments (e.g. questionnaires) informed by any formative primary research?  Q13a_formative  Q13b_focusgroups  Q13c_interviews  Q13e_research  Q13e_experts  Q13f_other | Yes, specify:   - Focus groups - Interviews - Previous surveys / research - Expert panel - Other:_______________   No / not specified | **Formative primary research** = research done prior to development of the study tools to inform the details of the study.  **Previous surveys / research** = explicit reference to previous survey studies or research literature as a basis for development of the data collection instruments. |
| 14) Were the data collection instruments (e.g. questionnaires) pre-tested?  Q14a_pretesting  Q14b_experts  Q14c_interviews  Q14d_pilotstudy  Q14e_yesbutnotspecified  Q14f_other  Q14g_na_qual | Yes, specify method:   - Expert review - Cognitive interviews with participants - Pilot study with participants - Not specified - Other:_______________   No / not specified  N/a - qualitative study | **Only indicate testing of “entire questionnaire instrument” in this section. *Any details on determining reliability and/or validity of specific composite scores/scales (e.g. Cronbach’s alpha, factor analysis, etc.) should NOT be answered in this question (they will be assessed in the risk-of-bias form).***  **Cognitive interviews =** qualitative technique where surveys are given to a limited number of participants (e.g. 10) from target population and they are asked to “talk aloud” about their thoughts as they go through each question. Usually conducted after questionnaire is developed, after any expert review, and before formal pilot testing to determine whether there are any significant design problems in the questions.  **Pilot study =** quantitative pre-testing of the questionnaire by administrating to a sample of the target population (e.g. 100-200 people) before the full study and analyzing responses to detect any problems/patterns of misinterpretation. Emulates procedures proposed in main study. |
| 15) What types of food premises were investigated?  Q15a_restaurants  Q15b_caterers  Q15c_butchershops  Q15d_delis  Q15e_grocery  Q15f_bakeries  Q15g_colleges  Q15h_lodging  Q15i_other  Q15j_notspecified | Restaurants  Caterers  Butcher shops  Delis  Grocery stores  Bakeries  Colleges / universities  Lodging facilities (e.g. hotels, motels)  Other, specify:_________  Not specified |  |
| 16) What types of food handlers were investigated?  Q16a_managers  Q16b_chefs  Q16c_workers  Q16d_servers  Q16e_other  Q16f_notspecified | Managers/owners  Chefs  Food workers (assistant cooks, preparers, other kitchen staff)  Servers/waiters  Other, specify:_________  Unspecified staff |  |
| 17) Was the study focused on food handlers/premises with any targeted sociodemographic characteristics?  Q17a_socio-demographics  Q17b_specifiy | Yes, specify:_____________  No / not reported | **Select options ONLY if the group was the main focus/target population of the study AS SPECIFIED BY THE AUTHORS**  E.g. ethnic restaurant operators, independent (vs. franchise/chain) establishments |
| 18) How were participants recruited?  Q18a_dialing  Q18b_agency  Q18c_database  Q18d_publicads  Q18e_onlineads  Q18f_other  Q18g_notspecified | Random-digit dialing  Marketing or survey research agency  Public or private database / list  Advertisements in public places  Advertisements online  Other, specify:_________  Not specified |  |
| 19) What was the final sample size?  Q19_samplesize | : ______________  Not reported | Report final sample size used for analysis. **For focus groups**, report number of groups conducted, average number pre group, and total number of participants across all groups. |
| 20) Was the study response rate reported?  Q20a_responserate  Q20b_responseratespecify | Yes, specify: ______________  No | If yes, specify response rate (percent) and number of participants included in sample out of number invited in text box. |
| 21) What outcomes were measured?  Q21a_knowledge  Q21b_attitudes  Q21c_behaviours  Q21d_beh_crosscontamination  Q21e_beh_menu  Q21f_beh_policies  Q21g_beh_database  Q21h_beh_other  Q21i_beh_notspecified  Q21j_beliefs  Q21k_role  Q21l_incidents  Q21m_numcustomers  Q21n_training  Q21o_tra_external  Q21p_tra_company  Q21q_tra_informal  Q21r_tra_other  Q21s_tra_notspecified  Q21t_interest  Q21u_other  Q21v_na_qual | Knowledge  Attitudes and risk perceptions  Behaviours and practices, specify:   - Actions taken to prevent cross-contamination - Allergen-specific menu, list, or other documentation - Allergy risk communication policies/practices - Maintenance of allergic customer database - Other:_______________ - Not specified   Beliefs about capabilities  Professional role and responsibilities  Prevalence of previous incidents  Number of customers with food allergies/intolerances  Training status, specify type:   - External/specific course - Company-delivered course - On-the-job/informal training - Other:_______________ - Not specified   Interest in future training/education  Other, specify:_________  N/a - qualitative study | **Knowledge =** An awareness of the existence of allergens/intolerances. E.g. measured from test/quiz scores or correct answers.  **Attitudes and risk perceptions =** broad category that might include constructs such as perceived threat of an allergic reaction (chance of occurrence, susceptibility, severity) and/or general concerns or beliefs about food allergies/food intolerances.  **Behaviours and practices =** includes self-reported or observed individual and restaurant practices to prevent cross-contamination, accommodate food allergies, and respond to any incidents.  **Training status** = prevalence of staff who have previously received food allergen/intolerance training.  **Beliefs about capabilities =** refers to self-confidence/comfort level/perceived easiness or difficulty in abilities to carry out a behaviour or action. Includes constructs such as perceived behavioural control, self-efficacy, locus of control.  **Professional role and responsibility** = e.g. perceived responsibilities of different stakeholder to prevent allergic reactions.  **Prevalence of previous incidents** = reported previous number of previous food allergy-related incidents.  **Number of customers with food allergies/intolerances** = reported previous or average number of customers reporting a food allergy/intolerance. |
| 22) What factors, if any, were evaluated for associations with one or more of the above outcomes?  Q22a_knowledge  Q22b_attitudes  Q22c_behaviours  Q22d_beliefs  Q22e_incidents  Q22f_numcustomers  Q22g_training  Q22h_interest  Q22i_sociodemographics  Q22j_premise  Q22k_other  Q22l_na | Knowledge  Attitudes and risk perceptions  Behaviours and practices  Beliefs about capabilities  Prevalence of previous incidents  Number of customers with food allergies/intolerances  Training status  Interest in future training/education  Sociodemographic variables  Food premise characteristics  Other, specify:_________  N/a – no associations investigated | **Socio-demographic variables** = includes variables such as age, gender, years of experience, education level, income level, race/ethnicity.  **Food premise characteristics** = includes variables such as independent vs. chain, number of employees/customers served, worker roles in the restaurant. |
| 23) Are any data of interest sufficiently reported to allow for possible meta-analysis?  Q23a_prevalencedata  Q23b_associationdata  Q23c_interventiondata  Q23d_nodata  Q23e_na_qual | Yes, prevalence outcomes  Yes, association outcomes  Yes, intervention outcomes  No  N/a - qualitative study | **Only answer this question with respect to associations relevant to the review question. Sufficient data includes:**  **Prevalence outcomes:**   - - Numerator **and** denominator, **or**   - Proportion + EITHER numerator or denominator   **Association/intervention outcomes:**  **Dichotomous:**   - - Numerator **and** denominator, **or** proportion + EITHER numerator or denominator for each group being compared, **OR** - Measure of association (e.g. odds ratio, relative risk) + EITHER a measure of variability (SE, CIs, variance) or an exact P-value   **Continuous:**   - - Mean, sample size, + EITHER a measure of variability (e.g. SD, CIs) or exact P-value/t-value in each group being compared **OR**   - Sample size and P-value/t-value from t-test **OR**   - Difference in means and a measure of variability (SD, SE, CIs, variance) **IR**   - Difference in means, sample size, + EITHER a common SD or an exact P-value /t-value   **Correlation coefficients:**   - Correlation coefficient and sample size or SE, **OR** p/t value and sample size (N). |
| **Additional questions for intervention studies** | | |
| 24) What type of intervention was investigated?  Q24a_course  Q24b_media  Q24c_other | Training session/course  Educational media or materials (e.g. fact sheets, pamphlets, posters)  Other, specify:________ |  |
| 25) What was the mode of intervention delivery?  Q25a_personind  Q25b_persongroup  Q25c_web  Q25d_print  Q25e_other | In-person (face-to-face):   - Individual - Group   Web-based/digital media  Print media (e.g. fact sheet, posters)  Other, specify:_____________ |  |
| 26) Was the intervention informed by any formative primary research?  Q26a_formative  Q26b_focusgroups  Q26c_interviews  Q26e_research  Q26e_experts  Q26f_other | Yes, specify:   - Focus groups - Interviews - Previous surveys / research - Expert panel - Other:_______________   No / not specified | **Formative primary research** = research done prior to development of the study tools to inform the details of the study.  **Previous surveys / research** = explicit reference to previous survey studies or research literature as a basis for development of the data collection instruments. |
| 27) Who, if anyone, was involved in the delivery of the intervention?  Q27_whoinvolved | Specify:________  Not reported  N/a | E.g. facilitators, instructors |
| 28) Describe the details of the intervention  Q28a_dose  Q28b_duration  Q28c_location  Q28d_notreported | Dose:_____________  Duration:________________  Location of delivery:___________  Not reported | **Dose:** refers to how often (and/or how much of) the intervention was applied (e.g. number of sessions conducted).  **Duration:** refers to how long the intervention was applied for.  Where possible provide units of measurement (e.g. total hours, weeks, etc.) |
| 29) Describe the content of the intervention  Q29_content | Specify:________  Not reported | E.g. what topics were covered. |
| 30) Describe the control group  Q30a_prepost  Q30b_control  Q30c_other  Q30d_notreported | Pre-post (before-and-after) design  Independent control group, specify details:_______________  Other, specify:_________  Not reported |  |
| 31) What was the length of participant follow-up?  Q31_followup | Specify:________  Not reported |  |
| 32) Additional comments:  Q32_comments | __________ |  |

**Risk-of-Bias Form**

| **Question / Excel code** | **Risk of bias** | **Definitions/additional notes** |
| --- | --- | --- |
| ***Are study participants likely to be representative of the target population?***  Bias_selection | - Yes (low risk of bias) - No (high risk of bias) - Unclear - Risk of bias is different across study outcomes, specify: | Describe whether selection of participants for the study was potentially not representative of the target population, including consideration of the response rate.  **Yes/low risk:** participant selection represented target population.  **No/high risk:** participant selection conducted in a way that does not represent target population.  **Unclear:** insufficient information provided to permit judgement. |
| ***Was measurement of the outcome variables valid and reliable?***  Bias_measure | - Yes (low risk of bias) - No (high risk of bias) - Unclear - Risk of bias is different across study outcomes, specify: | Reliability for outcomes reported as scales or composite measures of multiple questions/items should be measured for *internal consistency*, using Cronbach’s alpha (or comparable approach). Internal consistency indicates the extent that items compromising the scale are correlated (and measuring the same construct).  Validity of outcome variables (*construct validity*) should be assessed though *pre-testing* the questions with members of the target population (e.g. pilot study)  **Yes/low risk:** important reliability AND validity measurements are reported for each outcome of interest, as applicable for the given study. For internal consistency measures, Cronbach’s alpha values should be at least 0.60. If outcome was measured as single question/item, then internal consistency reliability is not applicable.  **No/high risk:** variables were not assessed for one or more important measures of validity and/or reliability, and/or scales were used with poor levels of validity/reliability (e.g. Cronbach’s alpha <0.6).  **Unclear:** insufficient information provided to permit judgement. If study indicates “validated” tool was used, but no details are provided on how validation was conducted, select this option.  ***Assessments should be made for each main outcome, as appropriate.*** |
| ***Were losses to follow-up (attrition) and exclusions from analysis reported?***  Bias_exclusions | - Yes (low risk of bias) - No (high risk of bias) - Unclear - Risk of bias is different across study outcomes, specify: | Evaluate the completeness of outcome data for each main outcome, including attrition (if applicable; for longitudinal studies) and exclusions from the analysis. Consider whether attrition and exclusions were reported, reasons for attrition/exclusions, and any re-inclusions in analyses performed by the review authors.  **Yes/low:** missing outcome measures were unlikely to bias the results (e.g. the proportion of missing data was small and unlikely to significantly affect the results).  **No/high:** missing outcome data was high and likely to bias the results.  **Unclear:** not specified in the paper (do not assume 100% follow up unless stated explicitly). Observations are missing and not explained.  ***Assessments should be made for each main outcome, as appropriate.*** |
| ***Did the authors report all intended outcomes?***  Bias_reporting | - Yes (low risk of bias) - No (high risk of bias) - Unclear | Evaluate how the possibility of selective outcome reporting was examined by the review authors, and what was found.  **Yes/low:** there is no evidence that outcomes were selectively reported (e.g. all relevant outcomes in the methods section are reported in the results section).  **No/high:** some important outcomes are omitted from the results.  **Unclear:** insufficient information provided to permit judgement. |
| ***Was the study free of other problems that could put it at a high risk of bias?***  Bias_other | - Yes (low risk of bias) - No (high risk of bias) - Unclear | Evaluate any important concerns about bias not addressed in the other domains in the tool (e.g. study funded by industry with concerns about sponsor involvement)  **Yes/low:** there is no risk of other biases (please specify details).  **No/high:** there is a risk of other biases (please specify details).  **Unclear:** possible risk of other biases but insufficient information provided to permit judgement (please specify details). |
| ***Overall risk-of-bias for each outcome (within-study summary assessment)***  Bias_overall | - Low - High - Unclear - Risk of bias is different across study outcomes, specify: | Consider if results are believable taking all study limitations into account. Consider issues that may limit your ability to interpret the results of the study.  **Low:** plausible bias unlikely to seriously alter the results. Low risk of bias for key domains.  **High:** plausible bias that seriously weakens confidence in the results. High risk of bias for key domains.  **Unclear:** plausible bias that raises some doubt about the results. Unclear risk of bias for key domains.  ***Assessments should be made for each main outcome, as appropriate.*** |

**Data Extraction Form:**

*Multiple forms should be submitted for each unique outcome reported in a study*

| **IMPORTANT NOTE:** Data to extract using this form must consist of one of the following formats, otherwise do not extract.   - **Only knowledge, training status, allergy incidents/customers, beliefs about capabilities (i.e. self-efficacy/confidence/comfort in food allergy practices), and behaviour/practices outcomes are of interest for data extraction** - **Data must be in a dichotomous or ordinal (e.g. never, sometimes, always) format. DO NOT extract continuous outcomes (e.g. mean scores)** - **Relevant dichotomous outcomes must report the numerator and denominator, or the proportion of respondents that answered each outcome PLUS either the numerator or denominator** - **If the outcomes are reported stratified by worker role/position (e.g. manager, chef, food worker, server), please extract the overall/total results only** | |
| --- | --- |
| **Question / Excel code** | **Options** |
| Specify the main topic of the outcome being extracted  Topic_category | Food allergy  Celiac disease/gluten-free meals  Other, specify:__________ |
| Specify the outcome category being extracted  Outcome_category | Knowledge  Behaviour/practices  Training status/policies *(specifically food allergy NOT general food safety)*  Number of allergic customers/previous allergy incidents |
| Specify the outcome variable description (as reported by the authors).  Outcome_description | :___________ |
| Specify how the outcome was measured (e.g. self-reported, correct/incorrect response, observed behaviour/practice)  Outcome_measure | :__________ |
| Specify the population (i.e. worker role/position) for the association outcome being extracted. If multiple groups are reported together, specify “multiple” then list each worker type.  Population | Owners  Managers  Chefs  Food workers  Servers/waiters  Other, specify:___________  Not specified |
| For the population group being extracted, specify the following demographic information. If not reported, indicate “not reported” in the cell.  Age_mean  Males_percent  Managers_percent | Mean age  Percent males (vs. females)  Percent managers/owners/operators compared to other work positions (e.g. food handlers, chefs, servers) |
| Extract quantitative outcome data in text boxes for each relevant category  Num_positive  Num_total  Response_options  Ordinal_category2-5 *(if more than 5 total categories, create additional columns as necessary)*  Ordinal_category2-5_des | **Dichotomous/ordinal data options**  Number positive (numerator)  Total number of respondents (denominator)  Specify response options (e.g. yes vs. no)  For ordinal data, specify above info for additional response categories as appropriate |
| Specify any additional comments | :___________ |

**Search Documentation**

***Search algorithms implemented in each database:***

| **Date** | September 21, 2017 |
| --- | --- |
| **Platform/Interface** | OvidSP |
| **Databases** | CAB Abstracts (1973-Present) |
| **Institution** | University of Guelph |
| **Search string:** | ((food AND allerg*) OR celiac OR coeliac) AND (restaurant* OR establishment* OR premise* OR cater* OR "food service" OR foodservice OR manager* OR hospitality)  in **Article title**  **OR**  in **Abstract**  **OR**  in **Descriptors** |
| **Hits** | 264 |
| **Limits** | None |

| **Date** | September 21, 2017 |
| --- | --- |
| **Platform/Interface** | Thomson Reuters Web of Science |
| **Databases** | Food Science and Technology Abstracts (1969-Present) |
| **Institution** | Ryerson University |
| **Search string:** | ((food AND allerg*) OR celiac OR coeliac) AND (restaurant* OR establishment* OR premise* OR cater* OR "food service" OR foodservice OR manager* OR hospitality)  in **Topic (title, abstract, keywords, descriptors, foreign title, commercial names)** |
| **Hits** | 284 |
| **Limits** | None |

| **Date** | September 21, 2017 |
| --- | --- |
| **Platform/Interface** | EBSCOhost |
| **Databases** | CINAHL (1937-Present) |
| **Institution** | Ryerson University |
| **Search string:** | ((food AND allerg*) OR celiac OR coeliac) AND (restaurant* OR establishment* OR premise* OR cater* OR "food service" OR foodservice OR manager* OR hospitality)  in **Title**  OR  in **Abstract**  OR  in **Subject Heading** |
| **Hits** | 50 |
| **Limits** | None |

| **Date** | September 21, 2017 |
| --- | --- |
| **Platform/Interface** | ProQuest |
| **Databases** | ProQuest Dissertations & Theses A&I‎ (1743-Present)  PsycINFO (1806-Present) |
| **Institution** | Ryerson University |
| **Search string:** | ((food AND allerg*) OR celiac OR coeliac) AND (restaurant* OR establishment* OR premise* OR cater* OR "food service" OR foodservice OR manager* OR hospitality)  in **Title**  **OR**  in **Abstract**  OR  in **Subject Heading** |
| **Hits** | 44 |
| **Limits** | None |

| **Date** | September 21, 2017 |
| --- | --- |
| **Platform/Interface** | Scopus |
| **Databases** | Scopus (1823-Present) |
| **Institution** | Ryerson University |
| **Search string:** | ((food AND allerg*) OR celiac OR coeliac) AND (restaurant* OR establishment* OR premise* OR cater* OR "food service" OR foodservice OR manager* OR hospitality)  in **Title**  **OR**  in **Abstract**  **OR**  **Key words** |
| **Hits** | 729 |
| **Limits** | None |

| **Date** | September 21, 2017 |
| --- | --- |
| **Platform/Interface** | PubMed |
| **Databases** | PubMed (1950-Present) |
| **Institution** | Ryerson University |
| **Search string:** | ((food AND allerg*) OR celiac OR coeliac) AND (restaurant* OR establishment* OR premise* OR cater* OR "food service" OR foodservice OR manager* OR hospitality)  in **All Fields**  **OR**  in **MeSH Terms** |
| **Hits** | 370 |
| **Limits** | None |

***List of articles used for search verification:***

| **Author** | **Title** | | **Source** | **Volume** | **Pages** | **Year** |
| --- | --- | --- | --- | --- | --- | --- |
| Virginia A. Stallings and Maria P. Oria (Editors) | Chp. 8: Managing Food Allergies in Retail, Food Service, Schools, Higher Education, and Travel Settings | | Finding a Path to Safety in Food Allergy: Assessment of the Global Burden, Causes, Prevention, Management, and Public Policy  Washington, DC: The National Academies Press | N/a | N/a | 2017 |
| Kronenberg,S. A. | Food Allergy Risk Management: More Customers, Less Liability | | *Journal of Foodservice Business Research* | 15 | 117-121 | 2012 |
| Madsen CB, Crevel R, Chan CH, Dubois AE, DunnGalvin A, Flokstra-de Blok BM, Gowland MH, Hattersley S, Hourihane JO, NÃ¸rhede P, Pfaff S, Rowe G, Schnadt S, Vlieg-Boerstra BJ. | Food allergy: stakeholder perspectives on acceptable risk | *Regulatory Toxicology and Pharmacology* | | 57 | 256-265 | 2010 |
| Worsfold,D. | Raising food allergy awareness of caterers in Wales | | *Nutrition and Food Science* | 38 | 417-421 | 2008 |

**List of Google search phrases:**

Food allergy knowledge food handlers

Food allergy attitudes food handlers

Food allergy beliefs food handlers

Food allergy behaviour food handlers

Food allergy management food handlers

Food allergy knowledge food service

Food allergy attitudes food service

Food allergy beliefs food service

Food allergy behaviour food service

Food allergy management food service

Food allergy knowledge retail

Food allergy attitudes retail

Food allergy beliefs retail

Food allergy behaviour retail

Food allergy management retail

Food allergy knowledge restaurant

Food allergy attitudes restaurant

Food allergy beliefs food restaurant

Food allergy behaviour restaurant

Food allergy management restaurant
