# Supplementary material for: A systematic review and meta-regression of the knowledge, practices, and training of restaurant and food service personnel toward food allergies and Celiac disease
Source: PLoS One. 2018 Sep 4;13(9):e0203496. doi: 10.1371/journal.pone.0203496 (PMC6122805; doi:10.1371/journal.pone.0203496)
Supplement: S3 File — (DOCX) [file pone.0203496.s003.docx]

**S3 File: Forest Plots for all Outcome Subgroups Not Reported in the Main Manuscript Document**

**Table of Contents**

[Correct response to various knowledge questions 3](#_Toc518463607)

[A food allergic reaction can occur if an individual touches a food item that contains allergens 3](#_Toc518463608)

[A food allergy reaction can cause death 4](#_Toc518463609)

[Arachis oil indicates that peanut is present in food production 5](#_Toc518463610)

[Customers with food allergies cannot safely consume a small amount of that food 6](#_Toc518463611)

[Food allergies are caused by the body’s negative reaction to proteins 7](#_Toc518463612)

[Fried foods can be dangerous for those with food allergies because cross-contact with other food proteins can occur 8](#_Toc518463613)

[High heat (e.g. cooking) cannot destroy food allergens 9](#_Toc518463614)

[If an individual is having an allergic reaction, serving them water is not an appropriate response strategy 10](#_Toc518463615)

[If an individual is having an allergic reaction, urgent medical attention is required 11](#_Toc518463616)

[Lactose intolerance is not the same as having a milk allergy 12](#_Toc518463617)

[Modern medicine cannot cure food allergies 13](#_Toc518463618)

[Removing an allergen from a prepared meal would not make it safe to eat 14](#_Toc518463619)

[The most effective treatment for a severe a food allergic reaction is injecting epinephrine 15](#_Toc518463620)

[Self-reported food allergen awareness 16](#_Toc518463621)

[Identification of major food allergens from a checklist 17](#_Toc518463622)

[Eggs 17](#_Toc518463623)

[Fish 18](#_Toc518463624)

[Milk/dairy 19](#_Toc518463625)

[Peanuts 20](#_Toc518463626)

[Shellfish 21](#_Toc518463627)

[Soy 22](#_Toc518463628)

[Tree nuts 23](#_Toc518463629)

[Wheat 24](#_Toc518463630)

[Able to identify at least three major allergens 25](#_Toc518463631)

[Identification of possible food allergy symptoms from a checklist 26](#_Toc518463632)

[Anaphylaxis 26](#_Toc518463633)

[Facial swelling 27](#_Toc518463634)

[Hives or rash 28](#_Toc518463635)

[Swelling of the throat or tongue 29](#_Toc518463636)

[Tingling in or around the mouth 30](#_Toc518463637)

[Trouble breathing 31](#_Toc518463638)

[Vomiting 32](#_Toc518463639)

[Practices and behaviours 33](#_Toc518463640)

[Allergen information is posted on website 33](#_Toc518463641)

[Allergens are identified on the menu or other documentation 34](#_Toc518463642)

[Allergen-free orders are recorded and verified with kitchen staff 35](#_Toc518463643)

[Food ingredient lists are available and/or checked for food allergens as necessary 36](#_Toc518463644)

[Separate allergen-free menu is provided 37](#_Toc518463645)

[Staff risk communication about food allergies with food allergic customers 38](#_Toc518463646)

[Training 39](#_Toc518463647)

[Respondent has previously received food allergy training 39](#_Toc518463648)

[Respondent is interested in future food allergy training 40](#_Toc518463649)

[Celiac Disease (CD) Knowledge and Practices 41](#_Toc518463650)

[Self-reported awareness of CD 41](#_Toc518463651)

[Self-reported awareness of gluten sensitivity 42](#_Toc518463652)

[Gluten-free foods are available 43](#_Toc518463653)

[Availability of gluten-free foods is indicated on the menu or other documentation 44](#_Toc518463654)

## Correct response to various knowledge questions

### A food allergic reaction can occur if an individual touches a food item that contains allergens

### A food allergy reaction can cause death

### Arachis oil indicates that peanut is present in food production

### Customers with food allergies cannot safely consume a small amount of that food

### Food allergies are caused by the body’s negative reaction to proteins

### Fried foods can be dangerous for those with food allergies because cross-contact with other food proteins can occur

### High heat (e.g. cooking) cannot destroy food allergens

### If an individual is having an allergic reaction, serving them water is not an appropriate response strategy

### If an individual is having an allergic reaction, urgent medical attention is required

### Lactose intolerance is not the same as having a milk allergy

### Modern medicine cannot cure food allergies

### Removing an allergen from a prepared meal would not make it safe to eat

### The most effective treatment for a severe a food allergic reaction is injecting epinephrine

## Self-reported food allergen awareness

## Identification of major food allergens from a checklist

### Eggs

### Fish

### Milk/dairy

### Peanuts

### Shellfish

### Soy

### Tree nuts

### Wheat

### Able to identify at least three major allergens

## Identification of possible food allergy symptoms from a checklist

### Anaphylaxis

### Facial swelling

### Hives or rash

### Swelling of the throat or tongue

### Tingling in or around the mouth

### Trouble breathing

### Vomiting

## Practices and behaviours

### Allergen information is posted on website

### Allergens are identified on the menu or other documentation

### Allergen-free orders are recorded and verified with kitchen staff

### Food ingredient lists are available and/or checked for food allergens as necessary

### Separate allergen-free menu is provided

### Staff risk communication about food allergies with food allergic customers

## Training

### Respondent has previously received food allergy training

### Respondent is interested in future food allergy training

## Celiac Disease (CD) Knowledge and Practices

### Self-reported awareness of CD

### Self-reported awareness of gluten sensitivity

### Gluten-free foods are available

### Availability of gluten-free foods is indicated on the menu or other documentation
