# Supplementary material for: A systematic review and meta-regression of the knowledge, practices, and training of restaurant and food service personnel toward food allergies and Celiac disease
Source: PLoS One. 2018 Sep 4;13(9):e0203496. doi: 10.1371/journal.pone.0203496 (PMC6122805; doi:10.1371/journal.pone.0203496)
Supplement: S1 Table — (DOCX) [file pone.0203496.s006.docx]

**S1 Table. Summary of the prevalence of restaurant and food service staff knowledge, practices, and training toward food allergies and Celiac disease across studies captured in this systematic review.**

| Outcome category / subgroup | No. of studies | Median (25^th^, 75^th^ percentile) of study estimates | Range of study estimates |
| --- | --- | --- | --- |
| **Correct response to various knowledge questions about food allergies^a^** |  |  |  |
| A food allergic reaction can occur if an individual touches a food item that contains allergens | 4 | 71% (57%, 74%) | 44-76% |
| A food allergy reaction can cause death | 10 | 89% (87%, 95%) | 51-97% |
| Arachis oil indicates that peanut is present in food production | 3 | 40% (27%, 50%) | 27-50% |
| Customers with food allergies cannot safely consume a small amount of that food | 14 | 80% (76%, 86%) | 46-93% |
| Food allergies are caused by the body’s negative reaction to proteins | 4 | 32% (22%, 49%) | 19-60% |
| Fried foods can be dangerous for those with food allergies because cross-contact with other food proteins can occur | 3 | 46% (26%, 81%) | 26-81% |
| High heat (e.g. cooking) cannot destroy food allergens | 12 | 73% (67%, 88%) | 45-93% |
| If an individual is having an allergic reaction, serving them water is not an appropriate response strategy | 9 | 66% (54%, 74%) | 40-83% |
| If an individual is having an allergic reaction, urgent medical attention is required | 5 | 87% (85%, 91%) | 65-99% |
| Lactose intolerance is not the same as having a milk allergy | 4 | 43% (28%, 45%) | 15-45% |
| Modern medicine cannot cure food allergies | 4 | 56% (40%, 64%) | 28-69% |
| Removing an allergen from a prepared meal would not make it safe to eat | 14 | 79% (72%, 85%) | 51-92% |
| The most effective treatment for a severe a food allergic reaction is injecting epinephrine | 5 | 61% (58%, 64%) | 5-76% |
| **Self-reported food allergen awareness** | 6 | 78% (51%, 93%) | 26-95% |
| **Identification of major food allergens from a checklist** |  |  |  |
| Eggs | 9 | 74% (62%, 72%) | 45-88% |
| Fish | 5 | 60% (56%, 66%) | 49-66% |
| Milk/dairy | 9 | 82% (81%, 85%) | 57-91% |
| Peanuts | 9 | 94% (93%, 95%) | 82-99% |
| Shellfish | 9 | 92% (89%, 93%) | 40-99% |
| Soy | 5 | 56% (54%, 61%) | 52-73% |
| Tree nuts | 7 | 83% (76%, 87%) | 59-91% |
| Wheat | 5 | 72% (70%, 78%) | 28-84% |
| **Able to identify at least three major food allergens** | 3 | 54% (25%, 56%) | 25-56% |
| **Identification of possible food allergy symptoms from a checklist** |  |  |  |
| Anaphylaxis | 4 | 72% (68%, 74%) | 66-75% |
| Facial swelling | 4 | 92% (89%, 95%) | 88-95% |
| Hives or rash | 5 | 95% (91%, 96%) | 89-98% |
| Swelling of the throat or tongue | 5 | 97% (91%, 97%) | 85-98% |
| Tingling in or around the mouth | 4 | 81% (77%, 83%) | 75-83% |
| Trouble breathing | 5 | 88% (83%, 88%) | 78-98% |
| Vomiting | 4 | 67% (64%, 71%) | 61-74% |
| **Food allergy practices and behaviours** |  |  |  |
| Allergen information is posted on website | 4 | 33% (26%, 41%) | 21-47% |
| Allergens are identified on the menu or other documentation | 12 | 32% (21%, 56%) | 3-83% |
| Allergen-free orders are recorded and verified with kitchen staff | 5 | 16% (12%, 49%) | 11-86% |
| Food ingredient lists are available and/or checked for food allergens as necessary | 9 | 61% (55%, 65%) | 22-93% |
| Policies or plans are in place to produce allergen-free meals | 17 | 62% (32%, 76%) | 1-95% |
| Policies or plans are in place to respond to food allergy emergencies | 7 | 25% (17%, 37%) | 0-58% |
| Separate allergen-free menu is provided | 3 | 25% (8%, 32%) | 8-32% |
| Staff risk communication about food allergies with food allergic customers | 5 | 44% (28%, 48%) | 24-87% |
| **Food allergy training** |  |  |  |
| Food allergy training is provided to food premise staff | 9 | 36% (32%, 69%) | 5-95% |
| Respondent has previously received food allergy training | 12 | 35% (26%, 44%) | 15-79% |
| Respondent is interested in future food allergy training | 7 | 61% (58%, 77%) | 48-80% |
| **Celiac disease (CD) outcomes** |  |  |  |
| Self-reported awareness of CD | 7 | 77% (37%, 89%) | 17-91% |
| Self-reported awareness of gluten sensitivity | 3 | 88% (9%, 89%) | 9-89% |
| Gluten-free foods are available | 4 | 84% (55%, 97%) | 38-100% |
| Availability of gluten-free foods is indicated on the menu or other documentation | 4 | 50% (35%, 72%) | 30-83% |
