# Supplementary material for: A systematic review and meta-regression of the knowledge, practices, and training of restaurant and food service personnel toward food allergies and Celiac disease
Source: PLoS One. 2018 Sep 4;13(9):e0203496. doi: 10.1371/journal.pone.0203496 (PMC6122805; doi:10.1371/journal.pone.0203496)
Supplement: S2 Table — (DOCX) [file pone.0203496.s007.docx]

**S2 Table. Summary of eight studies reporting on restaurant and food service staff self-efficacy towards food allergies.**

| Outcome type / study | Study country | Sample size | Findings and response options |
| --- | --- | --- | --- |
| **Self-efficacy in preparing or serving an allergen-free meal** |  |  |  |
| Ahuja and Sicherer (2007) [25] | US | 98 | 68% very or somewhat comfortable vs. comfortable, somewhat uncomfortable or very uncomfortable |
| Bailey et al. (2011) [26] | United Kingdom | 90 | 66% very comfortable, 16% somewhat comfortable, 13% comfortable, 6% somewhat uncomfortable, 0% very uncomfortable |
| Common et al. (2013) [27] | United Kingdom | 40 | 65% very comfortable, 25% somewhat comfortable, 10% comfortable, 0% somewhat uncomfortable, 0% very uncomfortable |
| Choi and Rajagopal (2013) [28] | US | 193 | 54% strongly agree, 39% agree, 6% neutral, 1% disagree, 0% strongly disagree (with statement about self-efficacy) |
| Wham et al. (2014) [29] | New Zealand | 124 | 57% very confident, 36% confident, 6% don’t know, 3% less confident, 0% not at all confident |
| Sogut et al. (2015) [30] | Turkey | 351 | 41% very comfortable, 9% somewhat comfortable, 38% comfortable, 8% somewhat uncomfortable, 4% very uncomfortable |
| Dupuis et al. (2016) [31] | US | 182 | 69% confident (scores of 85 or above on a 100-point linear scale) |
| Radke et al. (2016) [32] | US | 644 | 42% strongly agree, 49% agree, 2% neutral, 7% disagree, 0% strongly disagree (with statement about self-efficacy) |
| **Self-efficacy in responding to a food allergy emergency** |  |  |  |
| Choi and Rajagopal (2013) [28] | US | 193 | 18% strongly agree, 37% agree, 22% neutral, 19% disagree, 5% strongly disagree (with statement about self-efficacy) |
| Wham et al. (2014) [29] | New Zealand | 124 | 20% very confident, 44% confident, 21% don’t know, 12% less confident, 3% not at all confident |
| Dupuis et al. (2016) [31] | US | 179 | 54% confident (scores of 85 or above on a 100-point linear scale) |
